# Transcriptome and proteome responses in RNAlater preserved tissue of *Arabidopsis thaliana*

**DOI:** 10.1371/journal.pone.0175943

**Published:** 2017-04-19

**Authors:** Colin P. S. Kruse, Proma Basu, Darron R. Luesse, Sarah E. Wyatt

**Affiliations:** 1 Department of Environmental and Plant Biology, Ohio University, Athens, OH, United States of America; 2 Department of Biological Sciences, Southern Illinois University Edwardsville, Edwardsville, IL, United States of America; Pacific Northwest National Laboratory, UNITED STATES

## Abstract

Tissue preservation is a minimal requirement for the success of plant RNA and protein expression studies. The standard of snap-freezing in liquid nitrogen is not always practical or possible. RNAlater, a concentrated solution of ammonium and cesium sulfates, has become a standard preservative in the absence of liquid nitrogen. Here, we demonstrate the effectiveness of RNAlater in preserving both RNA and proteins in *Arabidopsis thaliana* tissues for use in RNAseq and LC-MS/MS analysis of proteins. While successful in preserving plant material, a transcriptomic and proteomic response is evident. Specifically, 5770 gene transcripts, 84 soluble proteins, and 120 membrane-bound proteins were found to be differentially expressed at a log-fold change of ±1 (P ≤ 0.05). This response is mirrored in the abundance of post-translational modifications, with 23 of the 108 (21.3%) phosphorylated proteins showing altered abundance at a log-fold change of ±1 (P ≤ 0.05). While RNAlater is effective in preserving biological information, our findings warrant caution in its use for transcriptomic and proteomic experiments.

## Introduction

For the success of transcriptomic and proteomic experiments sufficient quality and quantity of RNA or protein is essential. The ability to accurately preserve samples prior to extraction provides researchers flexibility with experimental design. Snap freezing in liquid nitrogen has been the standard technique for molecular and cell biologists and provides ideal preservation of proteins and nucleic acids for long periods when stored at -80°C. However, field experiments, remote regions of the world, and areas with insufficient infrastructure to allow for access to liquid nitrogen necessitate the use of fixatives [1,2].

RNAlater contains high concentrations of quaternary ammonium sulfates and cesium sulfates which denature RNases, DNases and proteases to prevent the degradation of RNA [3]. While RNAlater is ineffective in the preservation of fine anatomical and structural features [1], it has been shown to be effective in the preservation of nucleic acids [1,3,4]. RNAlater has been used to successfully preserve high quality DNA for up to 7 years from the date of sample collection and fixation in chimpanzee fecal material collected from remote habitats [1]. Parallel studies of RNAlater and formalin fixation on these fecal samples demonstrated RNAlater to be the superior fixative for the preservation of nucleic acids. In the past decade, studies on human tissues [3,5] and microbes [4] have shown that, in addition to nucleic acids, RNAlater is effective at preserving proteins. Saito et al. [4] analyzed microbial proteins and showed that more proteins were detected in RNAlater preserved samples than in the snap frozen controls. Bennike et al. [3,5] demonstrated protein and RNA integrity, and detection of post-translational modifications are not significantly impacted by RNAlater in human tissue samples.

RNAlater has been used for the preservation of plant material as well—especially space flown material grown aboard the international space station [2,6,7,8,9,10]. However, the structure of plant tissues present challenges for the use of fixatives, and how the cell wall impacts permeability of the fixative is unclear. Herein, we present, to our knowledge, the first full ‘omics level study performed to determine the ability of RNAlater to preserve tissue of *Arabidopsis thaliana*, the primary model organism for plants, for downstream ‘omics-level analyses.

## Results

### RNAlater treatment alters transcript levels in Arabidopsis seedlings

One caution with using RNAlater is the rate of its diffusion through tissues that could lead to uneven preservation of cells across tissues. Because RNAlater denatures transcriptional machinery, uneven preservation across tissues could lead to a transcriptomic or proteomic response. To determine if RNAlater treatment impacted transcript levels in *Arabidopsis thaliana*, etiolated seedlings were grown for 72 hours then either snap frozen in liquid N_2_, or Incubated for 12 h in RNAlater at room temperature followed by freezing at -80°C. RNAseq was performed and transcripts compared to assess differential gene expression. Between 17 and 30 million read pairs for each biological replicate were aligned to the TAIR10 assembly using STAR. For each replicate, at least 27,000 unique genes were detected using HTSeq, with at least 15,000 per replicate at greater than 5 counts per million. The ribosomal fraction was below 2% in all replicates.

**Fig 1:**
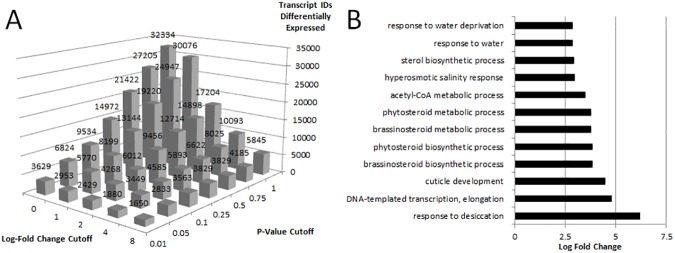
Number of differentially expressed genes identified by RNAseq analysis. A) the number of transcripts differentially expressed in RNAlater compared to liquid N_2_ shown by log-fold change (base two) and p-value cutoffs and B) the log fold enrichment of the GO terms for biological processes enriched for the 5770 transcript IDs differentially expressed at log-fold change ±1 (p ≤ 0.05). All reported GO terms are significant at a p-value of 0.05 or below.

Transcriptional activities in Arabidopsis were found to be modified by the method of preservation. At a log-fold change cutoff of ±1 (P ≤ 0.05), 5770 gene transcripts were differentially expressed (Fig 1A). More stringent log-fold change and significance cutoffs also identified transcripts that show differential regulation. We selected the 5770 as a robust number for subsequent analysis. A gene ontology (GO) enrichment analysis identified several upregulated categories indicating a transcript level response to osmotic stress (Fig 1B). Together, these results suggest that RNAlater preservation is not immediate in etiolated seedlings, and allows time for transcriptional response to osmotic stress caused by ammonium sulfates and cesium sulfates, resulting in potentially altered transcriptomes.

### RNAlater fixation allows for proteomics analysis

To determine if RNAlater fixation is sufficient to prevent proteolysis in Arabidopsis seedlings, etiolated plants were grown for 72 hours then either flash frozen in liquid N_2_ or submerged in RNAlater for 12 hours prior to protein extraction. Soluble and insoluble protein were separately collected from each sample and labeled with iTRAQ tags for Mass Spectrometry-based protein quantification. In total, 2,915 membrane proteins were identified in both conditions, whereas 2646 soluble proteins were identified in both the RNAlater and snap frozen samples. The detection of individual proteins is unaltered by the treatment, showing RNAlater to be effective for absolute detection of proteins.

### Differentially detected proteins

**Fig 2:**
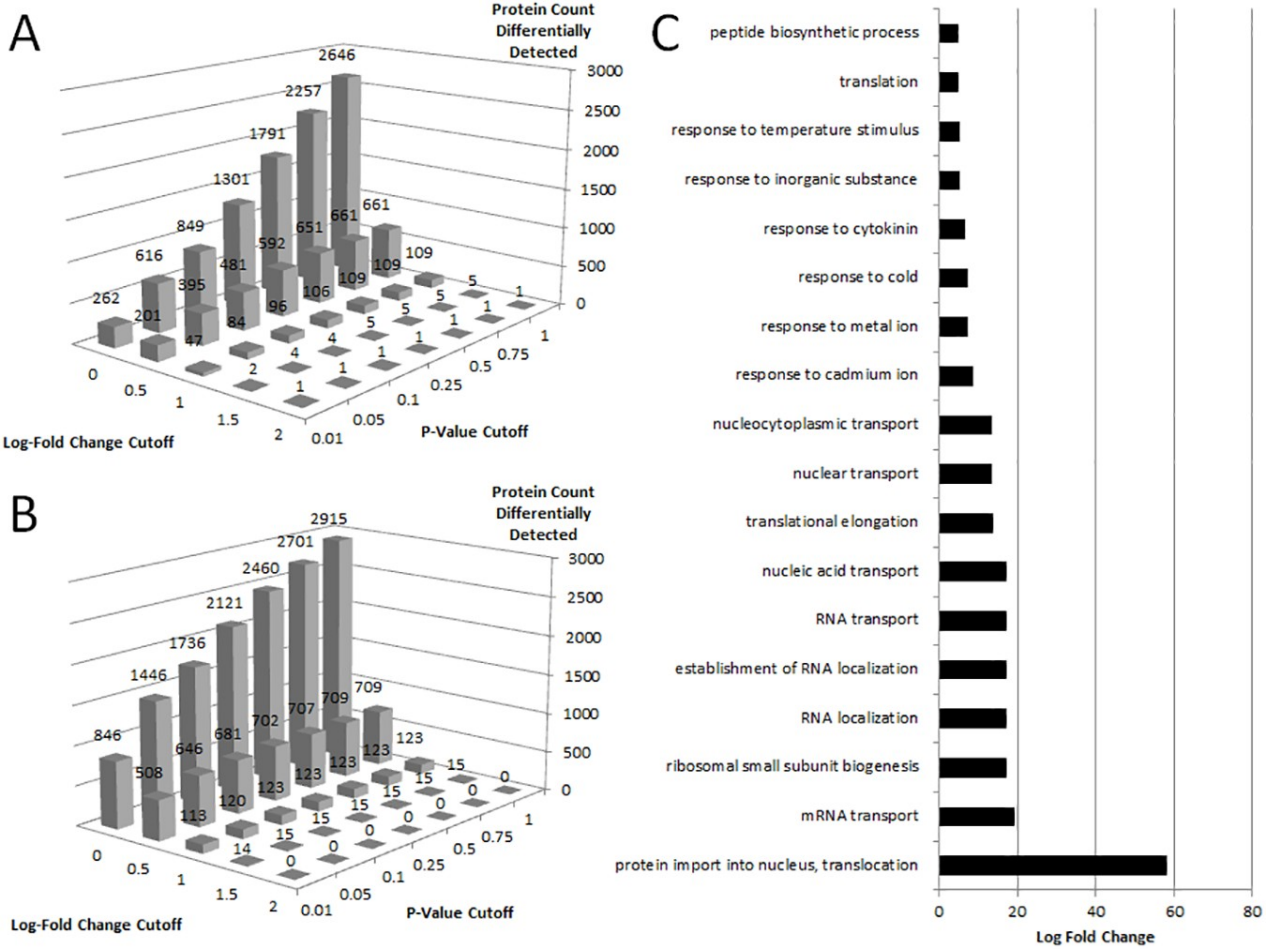
Proteomic analysis of RNAlater vs. Liquid N_2_ treated samples. The number of A) soluble proteins and B) membrane proteins considered significantly differentially abundant between treatments. C) The log-fold enrichment of the GO terms for biological processes for the proteins detected with a log-fold change ±1 (p ≤ 0.05). All reported GO terms are significant at a p-value of 0.05 or below.

RNAlater successfully preserved protein integrity to allow for reproducible detection of proteins, however RNAlater-fixed samples have altered protein expression levels. At a log fold change of ≥ ±1, 84 soluble proteins and 120 membrane-bound proteins were found to be differentially expressed (P ≤ 0.05) (Fig 2A & 2B). A GO term enrichment analysis of the proteins showed that, unlike the transcript analysis, the proteome did not show significant GO differences for categories comprising osmotic stress. However, the GO analysis did show significant differences in protein import into the nucleus (translocation), mRNA transport, ribosomal small subunit biogenesis, RNA localization, RNA/nucleic acid transport, translational elongation, and response to cadmium, metal, and cold, among others (Fig 2C).

### Post-translational modifications altered by RNAlater fixation

A growing focus of proteomics research is the detection of alterations in post-translational modifications. The resolution of our data allowed the determination of phosphorylation, oxidation, and deamidation of detected peptides. A total of 29 phosphorylated, 7060 oxidized, and 6001 deamidated peptides were detected in the soluble protein fractions with 79 phosphorylated, 3934 oxidized and 4035 deamidated peptides detected in the membrane fractions (Table 1).

**Table 1:** Number of peptides with altered post-translational modifications due to RNAlater.

Fraction	Post-translational modification	Total peptides identified with modification (#)	Peptides differentially modified*	
			#	% of total
**Soluble**	Phosphorylation	29	5	17.24
	Oxidation	7060	449	6.36
	Deamidation	6001	726	12.10
**Membrane**	Phosphorylation	79	18	22.78
	Oxidation	3934	301	7.65
	Deamidation	4035	318	7.88

*With significantly different abundances (p ≤ 0.05, LFC ±1 or greater)

In the membrane protein fraction, two modifications were found solely in the RNAlater preserved replicates (both deamidations). These modified peptides correspond to ROC4 and an ATP synthase beta-subunit (S1 Table). In the soluble fraction, 23 modified peptide states were found solely in the RNAlater preserved samples. These correspond to just two proteins—CRUCIFERIN 1 and 3 (S2 Table).

Few modifications seem to be selectively induced by RNAlater fixation, but the abundance of many protein modifications is altered by the treatment. Phosphorylated proteins show the greatest sensitivity to the treatment 21.3% of all phosphorylated peptides showing altered abundance. Oxidation and deamidation modifications are more tolerant of the treatment with 6.8% and 10.4% showing altered abundance of modified peptides, respectively.

## Discussion

RNAlater is a valuable fixative used for preservation of biological samples. The solution denatures proteases and RNases to preserve RNA and protein integrity for downstream analysis. While many studies have successfully utilized RNAlater as a preservative, few studies have directly compared the effects of this fixative on stability of RNA, protein and post-translational modifications. Bennike et al. [3] compared formalin, RNAlater and snap-freezing of human colon mucosal biopsies in a quantitative proteomics study which demonstrated RNAlater yielded the most similar results to liquid nitrogen. Additionally, the percentage of modified peptides detected was stable between RNAlater and snap-frozen samples. Specific amidation and oxidation events were not altered between liquid nitrogen and RNAlater fixation in human tissues [3,5]. Saito et al. [4] performed a comparison of snap-frozen samples to five preservative solutions (SDS-extraction buffer, ethanol, trichloroacetic acid, B-PER, and RNAlater) on the proteome integrity of cyanobacterium *Synechococcus* WH8102 and found that, after four weeks of preservation, RNAlater was the superior preservative and did not significantly alter the proteome compared to snap freezing in liquid nitrogen. In the 20 most abundant proteins in this microbe, the average relative abundance in RNAlater samples compared to liquid nitrogen samples was 1.01, indicating retention of protein integrity [4]. No equivalent control had been performed using plant tissues to examine the effects of RNAlater on plant proteomics nor to investigate transcriptomic modifications induced by the preservative.

Here, we show that fixation with RNAlater did not affect quality or quantity of proteins identified in Arabidopsis seedlings, consistent with previous studies using animal and bacterial tissue [3,4]. However, RNAlater treatment led to altered gene expression (Fig 1), protein expression (Fig 2) and abundance of post-translational modifications (Table 1). RNAlater had a substantial impact on the transcriptome, with 5770 transcripts differentially expressed at a log-fold change ±1 (Fig 1A). If the preserved tissue was responding to RNAlater, one would predict that the ammonium sulfates and cesium sulfates in the mixture would induce expression of genes related to salt stress. Indeed, genes associated with desiccation and osmotic homeostasis were the most enriched categories, suggesting that the transcript differences are a result of a cellular response to RNAlater (Fig 1B), potentially caused by an uneven preservation of cells as RNAlater diffuses across a tissue. Of the proteins detected, less than 1.4% of the membrane and soluble proteins, 120 and 84 respectively, were found to be altered between treatments at a log-fold change ±1 (Fig 2A and 2C). A GO enrichment analysis of the differentially abundant proteins did not indicate a salt stress response that could be attributed to the effects of RNAlater (Fig 2B and 2D). The absolute detection of post-translational modifications was unchanged by RNAlater, with the exception of peptides corresponding to four proteins—ROC4 (AT3G62030), an ATP synthase beta-subunit (AT5G08670), as well as CRUCIFERIN 1 and 3 (AT4G28520 and AT5G44120) (S1 and S2 Tables). Cruciferins are the most abundant seed storage proteins in Arabidopsis seedlings and have been implicated in response to oxidative stress [11]. The modifications may be a direct result of the oxidative stress produced by the ammonium salts and may indicate potential mechanism for the cruciferin protein’s ability to buffer seeds from oxidative stress. While the absolute detection of the remaining modifications was unaltered, the abundance of protein modifications was more sensitive to RNAlater fixation. Phosphorylation events were the most sensitive with 17.24% of the phosphorylated soluble proteins and 22.78% of the phosphorylated membrane proteins exhibiting an altered abundance of phosphorylated peptides. Deamidation and oxidation events were less sensitive to RNAlater (Table 1).

Interestingly, the drastic alterations in transcript levels were not mirrored in the proteome. While transcription can be quickly initiated in response to a change in environment, a change in translation would be slower and dependent on new transcripts. Thus, the rate of tissue infiltration allows a short-term alteration in transcription, but the RNAlater successfully inhibits the translational machinery before a substantial amount of protein is produced or degraded. The idea of a brief period of cellular response, prior to full fixation by RNAlater, is also supported by the observed changes in protein modifications (Fig 2).

Overall, these data suggest that researchers should use caution when selecting RNAlater as a fixative for plant omics-level experiments. Although it is convenient, depending on the sample, it may be unable to effectively halt transcriptional processes rapidly enough to prevent a transcriptomic response to the fixative. Theoretically, experimental controls, also fixed in RNAlater, would eliminate differential expression caused by the RNAlater-induced transcripts, preventing misidentification. However, some legitimate expression changes induced by experimental conditions could be obscured by RNAlater induction of the same genes.

## Methods

### Plant material

*Arabidopsis thaliana* (L.) Heyn. var Columbia seeds (Arabidopsis Biological Resource Center, Columbus, Ohio, USA) were used for all experiments. Forty-four aliquots of 0.016 ± 0.002 g of seeds (approximately 800–900 seeds per plate) were surface sterilized with 30% (v/v) bleach and 0.1% (v/v) Tween-20 for 20 min, washed with sterile water (5x), then dried onto 60 mm round filter paper to facilitate plating. Seeds were spread onto 60 mm round Petri plates containing 0.5 X MS media (Caisson) with 1% sucrose and 1% agar. Seeds were cold stratified then transferred to 22°C for germination and growth. After 72 hours, half of the samples were fixed in RNAlater for 12 hours prior to freezing at -80°C, while the remaining seedlings were snap-frozen in liquid nitrogen.

### RNA erxtraction and processing

Three biological replicates, one plate per replicate, were used for RNAseq analysis. RNA was extracted using an RNeasy Plant Mini Extraction Kit (Qiagen) according to standard protocols. RNA integrity (RIN ≥ 8) was verified using an Agilent 2100 Bioanalyzer. Paired-end sequence was obtained from an Illumina 2500 Hiseq using the Ribo-Zero rRNA Removal Kit prior to library preparation. Read pairs for each biological replicate were aligned to the TAIR10 assembly using STAR. Read counts were determined using HTSeq. Read counts were then analyzed to determine differential expression using the generalized linear model likelihood test within the EdgeR package. P-value was used to determine genes significantly expressed to maintain consistent statistics between protein and RNA data.

### Protein extraction

For extraction of soluble and membrane proteins, five plates were pooled for each replicate. Proteins were extracted according to Basu et al. [12]. The collected protein extract was centrifuged at 10,000 g for 60 min to precipitate the microsomal fraction. The resultant supernatant was used to precipitate soluble proteins by addition of 0.1 M methanolic ammonium acetate (Fisher), incubation at -20°C overnight then centrifugation at 16,000 g for 30 min. The pellet washed twice with 0.1M ammonium acetate (Fisher) solution, twice with 70% methanol and once with 80% acetone. The microsomal pellet was washed with sterile 160 mM sodium carbonate (Sigma) solution to remove any soluble protein contamination [13].

### Protein sample preparation

All samples from the soluble protein extraction were washed additionally with 1 x 0.1M ammonium acetate in methanol, 1 x 80% acetone and 1 x 70% methanol. The pellets were then re-dissolved with 75 μL of 8 M urea in 0.5 M TEAB (pH 8.5) and 4 μL of the solution was assayed for protein concentration using the CBX kit (GBioscience). 40 μg of each sample was reduced with 5 mM TCEP and alkylated with 10 mM iodoacetamide. Proteins were digested overnight at 37°C by adding 2.5 μg of trypsin (1:16 enzyme:substrate ratio), followed by addition of 1.25μg of trypsin for another 3 h. All were then dried to prepare for iTRAQ-labeling.

The membrane protein samples were centrifuged, and the supernatant assayed for protein using the CBX assay. To increase protein yield from the pellets, RapiGest (0.4%) was added to each pellet, and the samples incubated at 60°C for 30 min and centrifuged at 21,000 x g for 10 min. 40 μg of each sample was heated at 60°C, then cooled, reduced with 10 mM TCEP and alkylated with 20 mM iodoacteamide. The proteins were then digested by adding 2.5 μg of trypsin (1:16 enzyme:substrate ratio) overnight at 37°C; RapiGest was diluted to 0.1% with a 0.1M solution of TEAB; digestion was stopped after 3 h by acidifying to pH <2 and incubated for 2 h at 37°C to denature the RapiGest. Digested samples were centrifuged, the supernatant was decanted, and the samples were dried for iTRAQ8-labeling.

### iTRAQ labeling and LC MS/MS analysis

Equal amounts (40 μg) of both membrane and soluble, three replicates each, were digested with trypsin and labeled with a unique iTRAQ reagent according to the manufacturer’s protocol (AB SCIEX, Massachusetts, USA). During MS/MS iTRAQ reagents release characteristic reporter ions (114–119 m/z) for simultaneous relative quantification and identification of the proteins. Replicates for each sample were then pooled and dried. The pooled samples were reconstituted in 0.5% TFA, desalted by solid-phase extraction and dried. 100μg of each sample was fractionated using high pH reverse phase chromatography [14]. The fractions were combined into 12 final fractions for LC-MS/MS using an LTQ-Orbitrap Velos Pro mass spectrometer (ThermoFisher Scientific, Waltham, MA) coupled with a U3000 RSLCnano HPLC (ThermoFisher Scientific, Waltham, MA). Each pooled fraction was redissolved in 15 μL 5% acetonitrile, 0.1% formic acid, and 5 μL of each loaded per injection, onto a C18 trap column (PepMap100, 300μmID × 5mm, 5μm particle size, 100 Å; ThermoFisher Scientific, Waltham, MA) at a flow rate of 5 μL/min for 3.5 min with 2% acetonitrile, 0.1% formic acid. Peptide separation was then carried out on a C18 column (nanoAcquity CSH130, 25 cm ×75 μm, C18, 1.7 μm, 130 Å, Waters, Milford, MA) at a flow rate of 0.26 μL/min. Peptides were separated using the following gradient (mobile phase A, 0.1% formic acid; mobile phase B, 0.1% formic acid in 80:20 acetonitrile:water).

The mass spectrometer was operated in positive ionization mode. The MS survey scan was performed in the Orbitrap/FT cell from a mass range of 300 to 1700 m/z. The resolution set to 60 000 @ 400 m/z, and the automatic gain control (AGC) target set to 1, 000 000 ions with a maximum fill time of 10 ms and 1 μscan. HCD fragmentation was used for MS/MS, and the 10 most intense signals in the survey scan are fragmented. Detection was performed in the Orbitrap/FT cell with resolution set to 7,500, an isolation window of 3.0 m/z, a target value of 100,000 ions and a maximum fill time of 100 ms and 1 μscan. Fragmentation was accomplished with normalized collision energy of 40 and activation time of 0.1 ms. Dynamic exclusion was performed with a repeat count of 1 and exclusion duration of 60 s, and a minimum MS signal for triggering MS/MS is set to 10,000 counts.

### Protein data analysis

Mass spectrometry data analyses were automated using Mascot Daemon 2.5 (Matrix Science, London, UK). The raw data was processed to.mgf files using Proteome Discoverer 1.4 (Thermo Scientific, San Jose, CA, USA). Mascot (Matrix Science, London, UK) analysis of the MS/MS samples utilized the TAIR10 database (including target and decoy sequences, 35,386 entries), assuming trypsin digestion. Mascot searches used parent ion tolerance of 10.0 ppm and fragment ion mass tolerance of 0.06 Da. Fixed modifications were set to assume iTRAQ 8-plex modifications at the N-terminus and lysine residues and carbamidomethyl modifications at cysteine residues. Deamidation of asparagine and glutamine residues, oxidation of methionine residues and phosphorylation of serine, threonine and tyrosine residues were specified as variable modifications. The decoy database was used to adjust the FDR to 1%. Only proteins with at least two peptides with score > 20 were reported. The iTRAQ labeling efficiency was also evaluated and calculated to be 99.25% and 98.1% for soluble and membrane experimental set-ups respectively.

### Protein quantification and statistical analysis

Fold-changes were calculated using the average and standard deviation from each sample’s normalized ratios of iTRAQ labels. The relative quantification of each protein identified in the RNAlater and liquid N_2_ control were expressed as a ratio between the spectral counts of the isobaric tags. The p-value for testing significant differential abundance of proteins in each replicate was calculated using the Student’s t-test. Proteins with a p-value less than 0.05 and a log fold change greater than 1 or less than -1 were considered to be significantly different between experimental and control samples.

### GO enrichment analyses

GO enrichments were performed using the Gene Ontology Consortium’s web application [15,16] to determine the enrichment of biological process GO terms. For RNA, and total proteins, the list of genes or proteins submitted included those with a log-fold change ±1 (P ≤ 0.05). The analysis returned a list of biological processes with greater than expected frequency among the genes submitted. Gene annotation is based on prior studies and catalogued by the Gene Ontology Consortium’s Panther Classification System [17]. Gene ontology terms with a p-value ≤ 0.05 using the bonferroni correction for multiple comparisons were included.

## Supporting information

S1 Table Unique peptide modifications found in the membrane protein fraction. (DOCX)

S2 Table Unique peptide modifications found in the soluble protein fraction. (DOCX)

S1 File RNA, protein and post-translational modification data generated by the study. (XLSX)
