# Supplementary material for: Transcriptome and proteome responses in RNAlater preserved tissue of Arabidopsis thaliana
Source: PLoS One. 2017 Apr 19;12(4):e0175943. doi: 10.1371/journal.pone.0175943 (PMC5397022; doi:10.1371/journal.pone.0175943)
Supplement: S1 Table — (DOCX) [file pone.0175943.s001.docx]

**Supplemental Table 1.** Unique peptide modifications found in the membrane protein fraction

| Sequence Match (Peak matches) | Modified Peptide Sequence | Deamidated |
| --- | --- | --- |
| AT5G08670.1 (1) | GSITSVQAIYVPADDLTDPAPATTFAHLDATTVLSR | Q7 |
| AT3G62030.1 (1) | DFMIQGGDFTEGNGTGGISIYGAK | N13 |
