# Supplementary material for: Transcriptome and proteome responses in RNAlater preserved tissue of Arabidopsis thaliana
Source: PLoS One. 2017 Apr 19;12(4):e0175943. doi: 10.1371/journal.pone.0175943 (PMC5397022; doi:10.1371/journal.pone.0175943)
Supplement: S2 Table — (DOCX) [file pone.0175943.s002.docx]

**Supplemental Table 2.** Unique peptide modifications found in the soluble protein fraction

| Sequence Match (Peak matches) | Modified Peptide Sequence | Oxidized | Deamidated |
| --- | --- | --- | --- |
| AT4G28520.1 (2) | ALPLEVISNGFQISPEEAR |  | N9 |
| AT5G44120.3 (1) | QNAMVLPQWNANANAILYVTDGEAQIQIVNDNGNR |  | N30,N32 |
| AT5G44120.3 (1) | SGDTIATTPGVAQWFYNDGQEPLVIVSVFDLASHQNQLDR |  | Q35,Q37, N36 |
| AT5G44120.3 (8) | QNAMVLPQWNANANAILYVTDGEAQIQIVNDNGNR | M4 | N30,N32 |
| AT5G44120.3 (1) | QNAMVLPQWNANANAILYVTDGEAQIQIVNDNGNR | M4 | Q25,Q27 |
| AT5G44120.3 (1) | QNAMVLPQWNANANAILYVTDGEAQIQIVNDNGNR | M4 | Q27,N30, N32 |
| AT5G44120.3 (9) | QNAMVLPQWNANANAILYVTDGEAQIQIVNDNGNR | M4 | N32 |
